# The putative Tumor Suppressor VILIP-1 Counteracts Epidermal Growth Factor-Induced Epidermal-Mesenchymal Transition in Squamous Carcinoma Cells

**DOI:** 10.1371/journal.pone.0033116

**Published:** 2012-03-30

**Authors:** Katharina Schönrath, Andres J. Klein-Szanto, Karl H. Braunewell

**Affiliations:** 1 Signal Transduction Research Group, Institute of Neurophysiology, Charité University Medicine Berlin, Berlin, Germany; 2 Department of Pathology, Fox Chase Cancer Center, Philadelphia, Pennsylvania, United States of America; 3 Institute of Neurophysiology, Ruhr-University Bochum, Bochum, Germany; 4 Molecular and Cellular Neurosciences Laboratory, Department Biochemistry and Molecular Biology, Southern Research Institute, Birmingham, Alabama, United States of America; The University of Queensland, Australia

## Abstract

Epithelial-mesenchymal transition (EMT) is a crucial step for the acquisition of invasive properties of carcinoma cells during tumor progression. Epidermal growth factor (EGF)-treatment of squamous cell carcinoma (SCC) cells provokes changes in the expression of lineage markers, morphological changes, and a higher invasive and metastatic potential. Here we show that chronic stimulation with EGF induces EMT in skin-derived SCC cell lines along with the down-regulation of the epithelial marker E-cadherin, and of the putative tumor suppressor VILIP-1 (visinin-like protein 1). In esophageal squamous cell carcinoma and non-small cell lung carcinoma the loss of VILIP-1 correlates with clinicopathological features related to enhanced invasiveness. VILIP-1 has previously been shown to suppress tumor cell invasion via enhancing cAMP-signaling in a murine SCC model. In mouse skin SCC cell lines the VILIP-1-negative tumor cells have low cAMP levels, whereas VILIP-1-positive SCCs possess high cAMP levels, but low invasive properties. We show that in VILIP-1-negative SCCs, Snail1, a transcriptional repressor involved in EMT, is up-regulated. Snail1 expression is reduced by ectopic VILIP-1-expression in VILIP-1-negative SCC cells, and application of the general adenylyl cyclase inhibitor 2′,3′-dideoxyadenosine attenuated this effect. Conversely, EGF-stimulation of VILIP-1-positive SCC cells leads to the down-regulation of VILIP-1 and the induction of Snail1 expression. The induction of Snail is inhibited by elevated cAMP levels. The role of cAMP in EMT was further highlighted by its suppressive effect on the EGF-induced enhancement of migration in VILIP-1-positive SCC cells. These findings indicate that VILIP-1 is involved in EMT of SCC by regulating the transcription factor Snail1 in a cAMP-dependent manner.

## Introduction

Cell motility is a prerequisite for tumor progression and for invasive migration of carcinoma cells into surrounding tissue. In order to acquire a motile phenotype carcinoma cells undergo a dramatic morphological alteration, termed epithelial–mesenchymal transition (EMT), wherein they lose their epithelial characteristics and acquire the motility of mesenchymal cells [1]. In the case of many carcinomas, EMT-inducing signals, such as HGF, EGF, PDGF, and TGF-β, emanate from the tumor-associated stroma and activate a series of EMT-inducing transcription factors, including Snail, Slug, zinc finger E-box binding homeobox 1 (ZEB1), Twist, Goosecoid, and FOXC2. These transcription factors pleiotropically orchestrate the complex EMT program [2]. The loss of cell–cell contacts mediated by E-cadherin, an epithelial marker, is a typical hallmark of EMT [3]. The down-regulation of E-cadherin is common in squamous cell carcinomas (SCC) and is associated with an enhanced ability of invasion and/or metastasis and with a poor prognosis [4]–[6], reflective of its critical role in tumor progression. It is widely believed that the down-regulation of E-cadherin occurs through the transcriptional repression mediated by binding of transcriptional repressors, such as Snail1 (*SNAI1*) [7], [8], to E-box sequences in the proximal E-cadherin promoter [9]. The EMT program and the activation of Snail1 depends on a series of intracellular signaling networks and feedback loops involving ERK, MAPK, PI3K, and Akt signaling pathways [10]. In contrast, little is known about the involvement of cyclic nucleotide-mediated signaling pathways in EMT. These pathways are implicated in many biological processes that cooperate in organ development and differentiation of epithelial cells. The effects of cyclic adenosine monophosphate (cAMP) via protein kinase A (PKA) on changes in cell motility and via exchange protein activated by cAMP (EPAC) on cell migration [11] and integrin-mediated cell adhesion [12] are particularly important for tumor invasion. Intracellular cAMP concentrations are regulated by adenyl cyclases (AC), which use ATP to produce cAMP, and by phosphodiesterases (PDEs), which catalyze the degradation of cAMP to AMP [13].

Visinin-like protein 1 (VILIP-1, gene name *VSNL1*), a member of the family of neuronal calcium sensor proteins [14], modulates the levels of cyclic nucleotides, induces cell differentiation [15]–[17], and has recently been identified as a putative tumor migration suppressor gene [18], [19]. In esophageal cancer the reduced expression of VILIP-1 is correlated with invasive features, such as the depth of tumor invasion and local lymph node metastasis [20]. In aggressive non-small cell lung carinoma cell lines and primary tumors the loss of VILIP-1 expression is associated with a poor survival [21]. VILIP-1 is differentially expressed in chemically-induced murine skin squamous carcinomas of different degrees of aggressiveness. In an experimental model of murine SCC cell lines derived from these tumors it was demonstrated that the ectopic expression of VILIP-1 in two VILIP-1 non-expressing, high grade SCC lines increased cAMP levels, leading to a diminished MMP-9 and RhoA activity together with a significant reduction in the invasive properties of the carcinoma cells [18]. VILIP-1 expression was further shown to decrease the expression of fibronectin-specific integrin subunits α5 and αv that contributed to cell adhesion, cell migration, and invasiveness of highly invasive SCC cell lines [19]. Recently, we demonstrated that the tumor invasion suppressing effect of VILIP-1 in mouse skin SCCs exclusively depends on cAMP levels, but not on cGMP levels, and that both cAMP-effectors, PKA and EPAC, are involved in the reduction of the migratory ability of SCC cells [22]. Here, we set out to investigate, whether and how VILIP-1-enhanced cAMP-signaling may be involved in EMT in SCC.

## Materials and Methods

### Material

FSK (adenylyl cyclase activator Forskolin), 8Br-cAMP, DDA (2′,5′-dideoxyadenosine, general AC inhibitor) EGF and TGFβ for cell stimulation experiments were obtained from Sigma (St. Louis, MO, USA), Tocris (Bristol, UK) and Calbiochem (San Diego, CA, USA). Cell culture reagents were obtained from Gibco-Invitrogen (San Diego, CA, USA). Unless otherwise specified, all other reagents were purchased from Sigma and Roth (Karlsruhe, Germany).

### Antibodies

Rabbit polyclonal antibodies, raised against recombinant VILIP-1 protein, were affinity-purified on corresponding glutathion-S-transferase (GST)-tagged fusion proteins, immobilized on N-hydroxysuccinimide ester coupled agarose colums (Bio-Rad, Hercules, CA, USA) as previously described [23]. Polyclonal rabbit anti E-cadherin (gp184) antibodies were kindly provided by Otmar Huber and described previously [24]. Polyclonal rabbit anti integrin α5 antibodies were purchased from Chemicon (Temecula, CA, USA) and monoclonal antibodies against β-actin (sc-81178) and HRP-labeled secondary antibodies were purchased from Santa Cruz Biotechnologies (Santa Cruz, CA, USA).

### Cells and culture method

Murine skin squamous cell carcinoma cell lines CC4A and CC4B, CH72 and CH72T3 were described previously [17]. CC4A and CC4B were derived from the same tumor. When injected s.c. into nude mice, CC4A gave rise to a high-grade SCC or spindle cell carcinoma (or SCC IV), whereas CC4B gave rise to a well-differentiated, less aggressive, and low-grade SCC (SCCII). CH72 also gave rise to a low-grade SCC after s.c. inoculation, and CH72T3 is a subcloned cell line obtained by *in vivo* passaging of CH72 into nude mice, which resulted in a high-grade SCC. Cells were grown in DMEM (GIBCO) plus FCS (10%), L-glutamine (2 mM) and penicillin/streptomycin (100 µg/ml).

### Growth factor treatment

CC4B and CH72 cells were plated in standard DMEM in 24-well or 6-well dishes, respectively. 24 h after plating and 8 h prior to treatment with EGF or TGFβ medium was exchanged to low FCS (1%) DMEM to basal the cells. Cells were treated for 72 h with the indicated concentrations of growth factors and afterwards lysed for Western blot or RT-PCR analysis. To compare morphological changes cells were fixed and images were taken with a Leica inverted microscope at a 200× magnification. The migratory capacity of the cells after growth factor treatment was analyzed in *in vitro* wounding assays over 24 h. In indicated cases agents increasing or decreasing cAMP concentrations were added 24 h before cell lysis or before wounding the cell monolayer.

### Transfection

CC4A and CH72T3 were transfected with VILIP-1-GFP-vector or empty-GFP-vector [26] whereas CC4B and CH72 were transfected with VILIP-1-siRNA or scrambled siRNA using Optimem and lipofectamin 2000 (Invitrogen) following the manufacturer's instructions. VILIP-1-siRNA (antiVILIP1_1: sense r(AGC CGU UAG UCU GAA UUA A)dTdT, antisense r(UUA AUU CAG ACU AAC GGC U)dAdA; antiVILIP1_2: sense r(CAA AGA UGA CCA GAU UAC A)dTdT, antisense r(UGU AAU CUG GUC AUC UUU G)dAdA; antiVILIP1_3: sense r(GUG CGA CAU UCA GAA AUG A)dTdT, antisense r(UCA UUU CUG AAU GUC GCA C)dAdA) was used as a cocktail of three siRNA oligos (150 ng of each per transfection) directed against the coding region of VILIP-1 and was purchased from Qiagen (Hilden, Germany).

### Western blot analysis

Cultured cells were homogenized in an appropriate volume of homogenization buffer (25 mM Tris, 150 mM NaCl, pH 7.5, containing the protease inhibitors benzamidine (1 mM), phenylmethylsulfonylfluoride (0.1 mM)). Nuclei and debris were removed by centrifugation at 1.000 g for 5 min, protein concentrations were measured using BCA assay (Pierce, Rockford, IL, USA) and 40 µg protein of each sample was applied to 5–20% gradient SDS-PAGE. To analyze the expression level of VILIP-1, E-cadherin, integrin α5 and β-actin separated proteins were blotted on a PVDF membrane. The membrane was blocked with 5% milk powder in TBST (25 mM Tris, 150 mM NaCl, pH 7.5, 1% Tween 20) for 1 h at RT and afterwards incubated with the primary antibodies at 4°C overnight as previously described. After washing three times with TBST, secondary antibodies were applied for one hour at RT. Unbound antibodies were removed and the detected protein was visualized in a dark chamber using Western Lightning reagents (PerkinElmer Life Sciences, Boston, MA USA) and Hyperfilm (Amersham, UK).

### RT-PCR

PCR primers were designed that selectively amplify cDNA encoding Snail1, VILIP-1 or GAPDH and synthezised by Invitrogen (Carlsbad, CA,USA) (Snail1: sense AGG ACG CGT GTG TGG AGT, antisense GGAGAATGG CTT CTC ACC AG; VILIP-1: sense ATG GGG AAR CAG AAT AGC AAA C, antisense TCA TTT CTG MAT GTC KCA CTG CA; GAPDH: sense ACC ACA GTC CAT GCC ATC AC, antisense TCC ACC ACC CTG TTG CTG TA; K, M, R indicate mixed bases used to obtain species-independent primers). RT-PCR experiments were performed 3 times using total RNA from SCC-lines. Total RNA was extracted using RNeasy Mini-Kit (Quiagen, Hilden, Germany) and reverse transcribed using Oligo(dT) primers and SuperScript III First-Strand-Kit (Invitrogen, San Diego, CA, USA). PCR was performed using 0.2 µM of each primer, PCR buffer, 0.2 mM dNTP-Mix, 2 mM MgCl_2_, and 1 U taq polymerase (Invitrogen, San Diego, CA, USA) and DEPCH_2_O in a 50 µl reaction mix. 35 cycles of amplification were performed for each sample. For each primer pair the reaction was also carried out in absence of reverse transcriptase to ensure that there is no DNA contamination.

### *In vitro* wound assay

Cells grown in standard medium (2×10^5^ cells/well) were plated in 24-well plates. Cells were either grown in low FCS (1%) medium for 8 h and then treated with 10 ng/ml EGF in low FCS (1%) medium for 72 h before wounding or were transfected with VILIP-1-siRNA or the corresponding control 72 h before wounding and grown to confluence. Cells were placed in low FCS (1%) medium in order to basal the cells prior to growth factor treatment and to minimize cell proliferation. A wound was created by scratching the cell monolayer using a sterile 200 µl pipette tip. The wound was marked and 24 h after wounding cells were fixed and pictures were taken at a 200× magnification with a Leica inverted microscope and at least eight representative fields for each condition were analyzed. Cell migration was quantified by counting the number of cells/field.

### Statistical analysis

Statistical analysis was performed using unpaired, two-sided Student's t-test for samples of unequal variance (Welch test). Values were accepted as significant when p was less than 0.05 (*), less than 0.01 (**) or less than 0.001 (***). All error bars represent standard deviations.

## Results

### Epithelial-mesenchymal transition (EMT) in squamous cell carcinoma (SCC)

**Figure 1:**
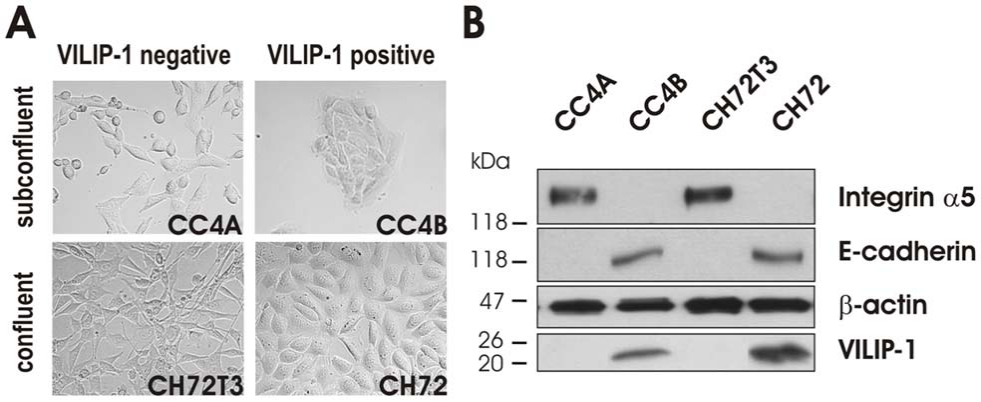
EMT-related differences in the characteristics of VILIP-1-positive and VILIP-1-negative SCC. **A**. Differences in morphology and in the formation of cell-cell contacts between VILIP-1-negative, aggressive CC4A and CH72T3 cells and the VILIP-1-positive, less aggressive CC4B and CH72 cells. **B**. Western Blot analysis showing the reciprocal expression levels of the adhesion molecules E-cadherin and integrin α5 in the VILIP-1-negative and -positive cell lines. As control for protein loading the β-actin levels were examined.

When we compared the morphology of cultures of the less aggressive, VILIP-1-positive skin cancer cells (CC4B and CH72) with the more aggressive, VILIP-1-negative skin cancer cells (CC4A and CH72T3), obvious morphological differences were noticed. Less aggressive, VILIP-1-positive skin cancer cells (CC4B and CH72) were well organized, tightly packed and formed clustered, cobblestone-like structures, typical of epithelial cells and suggestive of strong cell–cell adhesion. In contrast, aggressive, VILIP-1-negative skin cancer cells (CC4A and CH72T3) showed the mesenchymal morphological phenotype, including cell shape elongation and scattering of cells, which is suggestive of reduced cell–cell adhesion and increased cell motility (Fig. 1A). Since cell-cell adherens junctions of epithelial cells are formed by E-cadherin molecules, we assessed the cellular expression levels of E-cadherin in VILIP-1-negative and VILIP-1-positive SCCs. Immunoblotting showed that the expression of E-cadherin was down-regulated in VILIP-1-non-expressing cell lines CC4A and CH72T3, compared to VILIP-1-expressing cell lines CC4B and CH72. In contrast the integrin receptor subunit α5, mediating cell-matrix adhesion, was up-regulated in VILIP-1-non-expressing cells (Fig. 1B). The loss of the epithelial, cell type-specific morphology, the loss of E-cadherin expression and the associated reduction of cell-cell adherens junctions are hallmarks of EMT. These results suggest that aggressive SCCs must have undergone EMT while losing VILIP-1-expression.

### Growth factor-induced EMT: changes in cell morphology and expression of marker proteins in SCC

**Figure 2:**
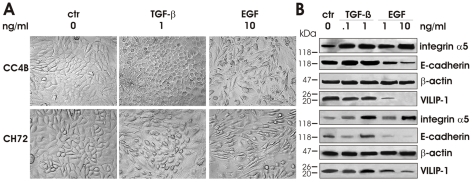
Effects of growth factor treatment in VILIP-1-positive, less aggressive SCC. **A**. Changes in cell shape and cell-cell adhesion following treatment of CC4B and CH72 with 1 ng/ml TGFβ or 10 ng/ml EGF. **B**. Western Blot analysis showing the changes in the expression level of integrin α5, E-cadherin and VILIP-1 following treatment with TGFβ (lanes 2 and 3, 0.1 and 1 ng/ml) or EGF (lanes 4 and 5, 1 and 10 ng/ml). As control for protein loading the β-actin levels were examined. Representative pictures out of three independent experiments are shown.

Growth factors, especially TGFβ and EGF, have been shown to induce EMT along with the down-regulation of various epithelial markers, including E-cadherin, in SCC [25]. We thus examined whether the stimulation of VILIP-1-positive CC4B and CH72 cells with different concentrations of TGFβ or EGF promotes EMT-like morphological changes and corresponding alterations in expression levels of VILIP-1, E-cadherin and integrin α5. When TGFβ-treated CC4B and CH72 cells were compared to untreated cells (control), they appeared rounded in cell shape (Fig. 2A, second panel) and immunoblotting revealed increased integrin α5 expression levels and slightly reduced VILIP-1 expression levels (Fig. 2B, lanes 2 and 3). In terms of E-cadherin protein levels, the induced alterations were not consistent between the two cell lines. Contrary to expectations, in CC4B cells E-cadherin protein levels cells seemed increased following TGFβ treatment (Fig. 2B lanes 2 and 3, upper panel) and correspondingly CC4B cells did not show scattering (Fig. 2A, upper row, second panel). However, CH72 cell responded in an inhomogeneous manner to TGFβ treatment. Stimulated CH72 monolayers exhibited areas of scattered cells (Fig. 2A, lower row, second panel) and stimulation with 0.1 ng/ml TGFβ slightly reduced E-cadherin protein levels in 2 of 3 repeats, whereas stimulation with 1 ng/ml TGFβ slightly increased E-cadherin expression (Fig. 2B, lower panel). In summary, TGFβ treatment had only moderate effects on VILIP-1 protein levels, did not alter or even tend to increase E-cadherin protein levels and did not lead to widespread cell scattering or cell shape elongation. Hence, TGFβ did not cause a shift from the VILIP-1-positive, less aggressive phenotype to the VILIP-1-negative, aggressive phenotype of SCC. By comparison, EGF treatment at 10 ng/ml resulted in a more obvious cell shape elongation and scattering of CC4B and CH72 cells (Fig. 2A third panel). Immunoblotting showed that EGF at 10 ng/ml caused down-regulation of E-cadherin in both carcinoma lines, which was consistent with the observed EGF-induced cell morphological changes. Interestingly, the expression of VILIP-1 was also clearly down-regulated in both cell lines in response to stimulation with 10 ng/ml EGF. Stimulation with 1 ng/ml EGF did produce a less pronounced down-regulation of E-cadherin and VILIP-1. Integrin α5 was up-regulated with increasing EGF concentrations in both cell lines (Fig. 2B, lanes 4 and 5). Collectively, the stimulation at the higher EGF concentration (10 ng/ml) induced clear EMT-like changes, and resulted in a shift in the morphology and the protein expression of VILIP-1-positive, less aggressive SCCs towards the phenotype of VILIP-1-negative, more aggressive SCCs shown in Fig. 1.

### Effect of the modulation of VILIP-1-expression on integrin α5 and E-cadherin expression

**Figure 3:**
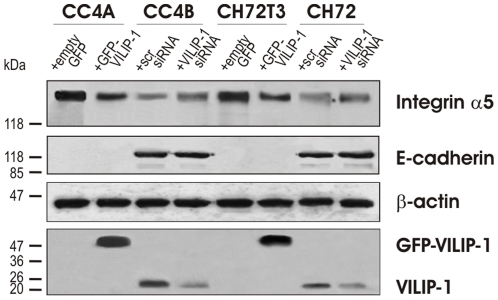
Effect of modulation of VILIP-1 levels on the expression of EMT markers. Western Blot analysis showing decreased expression of integrin α5 and unchanged E-cadherin expression following ectopic expression of VILIP-1 (+GFP-VILIP-1: 47 kDa) in VILIP-1-negative SCCs, CC4A and CH72T3. Increased expression of integrin α5 and unchanged E-cadherin expression is observed following siRNA knock down of VILIP-1 in VILIP-1-positive SCCs, CC4B and CH72, in comparison to the corresponding control-treated SCC (endogenous VILIP-1: 22 kDa, compare lanes 3 and 4, and lanes 7 and 8). As control for protein loading the β-actin levels were examined.

In a previous study it has been shown that the knock down of VILIP-1-expression caused an increase in the expression level of integrin α5 and αv in skin SCC [19]. Similarly, we found an increased expression of integrin α5, while VILIP-1 expression was down-regulated following EGF-stimulation. To determine whether the loss of VILIP-1 also affected the expression of E-cadherin, we transfected VILIP-1-negative SCCs with GFP-VILIP-1 or empty GFP-vector as control, and VILIP-1-positive SCCs with VILIP-1-specific siRNA or scrambled siRNA as control for 72 h respectively and assessed protein levels of E-cadherin (Fig. 3). Immunoblotting confirmed that integrin α5 expression is inversely regulated by VILIP-1 (down-regulation in CC4A by 42%, in CC72T3 by 44%; up-regulation in CC4B by 37%, in CH72 by 55%). In contrast, no effect of either VILIP-1 overexpression in VILIP-1-negative SCCs CC4A and CH72T3 or VILIP-1 knock down in VILIP-1-positive SCCs CC4B and CH72 on E-cadherin expression was observed, indicating that E-cadherin and VILIP-1 are independently down-regulated by EGF during EMT.

### Expression levels of Snail1 mRNA in VILIP-1-positive and VILIP-1-negative SCC and effect of EGF

**Figure 4:**
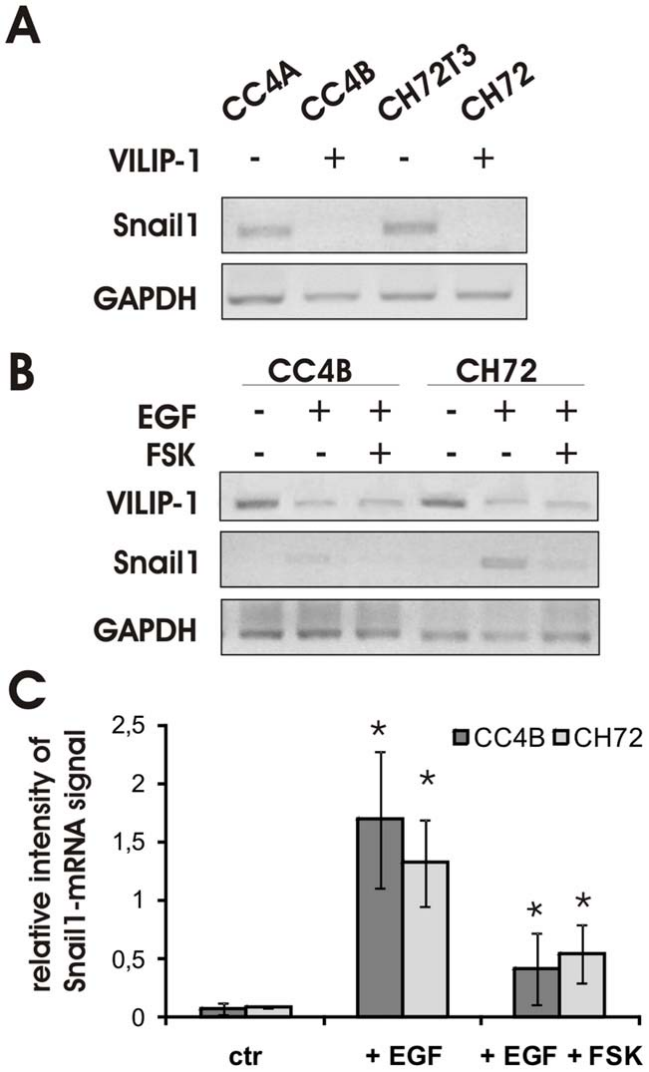
Expression levels of Snail1 mRNA in VILIP-1-positive and VILIP-1-negative SCC and effect of EGF. **A**. RT-PCR analysis showing the expression of Snail1 in VILIP-1-negative, aggressive CC4A and CH72T3 cells using GAPDH mRNA expression as internal control. **B**. Effect of EGF stimulation (lanes 2, 3 and 5, 6) and of additional forskolin treatment (lanes 3 and 6) on the expression of Snail1 mRNA in VILIP-1-positive, less aggressive CC4B and CH72 cells. For comparison the expression of VILIP-1 mRNA and GAPDH mRNA is shown. Representative pictures of three independent experiments are shown. **C**. Densitometry of RT-PCR analysis in B, lanes 1 to 6. Intensity of Snail1 bands was normalized to the intensity of GAPDH control PCR bands (CC4B+EGF: p = 0.039, CH72+EGF p = 0.029, CC4B+EGF+FSK: p = 0.04, CH72+EGF+FSK: p = 0.047). Bars represent the mean of three experiments. Error bars indicate standard deviations. Asterisks indicate the level of significance.

Snail1 (*SNAI1*), a member of the slug/snail family of transcriptional repressors [8], is one of the several transcriptional factors that can suppress E-cadherin gene expression in squamous cell carcinoma and is a potent inducer of EMT [26], [27]. Accumulating evidence indicates that the EGFR family and its downstream signaling pathways, the PI3K–Akt- and MEK–ERK pathway, regulate the expression of Snail1 [28]–[31], suggesting Snail1 as a candidate repressor for the down-regulation of VILIP-1 and E-cadherin expression in response to stimulation with EGF in mouse skin SCC. To verify this hypothesis we first determined the expression of Snail1 in the aggressive and less aggressive SCC cell lines. RT-PCR analysis showed that Snail1 mRNA is solely detectable in VILIP-1-negative aggressive SCC cell lines (Fig. 4A). However, following EGF stimulation and subsequent EMT-induction, Snail1 was up-regulated in VILIP-1-positive SCC cell lines (Fig. 4B). Interestingly, the induction of Snail1 expression in response to EGF was diminished in the presence of elevated cAMP following forskolin (FSK) stimulation (Fig. 4B), indicating a novel role of cAMP-signaling in EMT. Quantification of the RT-PCR showed that the EGF-induced increase of Snail1 mRNA was statistically significant compared to control (Fig. 4C; CC4B+EGF: p = 0.039, CH72+EGF: p = 0.029). Co-treatment with EGF and forskolin significantly attenuated the EGF-induced increase of Snail1 mRNA (Fig. 4C; CC4B+EGF+FSK: p = 0.04, CH72+EGF+FSK: p = 0.047).

### The effect of the modulation of VILIP-1-expression on Snail1-expression depends on cAMP-signaling

**Figure 5:**
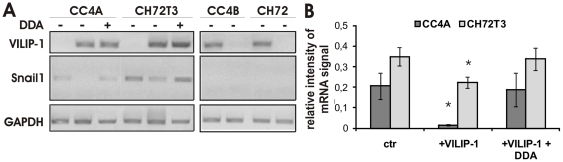
The effect of modulation of VILIP-1-expression and cAMP-signaling on Snail1 mRNA levels. **A**. RT-PCR analysis showing the suppression of Snail1 mRNA by ectopic expression of VILIP-1 in VILIP-1-negative, aggressive CC4A and CH72T3 cells (lanes 2, 3 and 5, 6) and the blocking of this effect by application of DDA (500 µM) (lanes 3, 6). siRNA knock down of VILIP-1 in VILIP-1-positive less aggressive CC4B and CH72 cells caused no alteration of the expression level of Snail1 (lanes 8, 10). **B**. Densitometry of RT-PCR analysis in A, lanes 1 to 6. Intensity of Snail1 bands was normalized to the intensity of GAPDH control PCR bands (CC4A+VILIP-1: p = 0.035, CH72T3+VILIP-1 p = 0.037). Bars represent the mean of three experiments. Error bars indicate standard deviations. Asterisks indicate the level of significance.

Since the expression of VILIP-1 increases intracellular levels of cAMP in skin SCC [18], we analyzed the effect of VILIP-1 and cAMP-signaling on Snail1 mRNA levels. Following transfection of VILIP-1-negative SCCs with GFP-VILIP-1 or empty GFP-vector as control, and VILIP-1-positive SCCs with VILIP-1-specific siRNA or scrambled siRNA as control for 72 h, we found that knock down of VILIP-1-expression did not affect Snail1 mRNA expression. In contrast, ectopic expression of VILIP-1 in the aggressive, VILIP-1-negative cell lines CC4A and CH72T3 reduced Snail1 mRNA levels (Fig. 5A). The reduction of Snail1 mRNA was statistically significant (Fig. 5B; CC4A: p = 0.035, CH72T3 p = 0.037) and could be blocked by the application of the general adenylyl cyclase inhibitor DDA for 24 h before lysis (Fig. 5A, 5B lanes 2 and 4 versus 3 and 6, respectively), demonstrating that cAMP-signaling plays an important role for the VILIP-1 effect on Snail1 expression.

### Involvement of cAMP-signaling in the VILIP-1-siRNA- or EGF-induced migration of SCC cells

**Figure 6:**
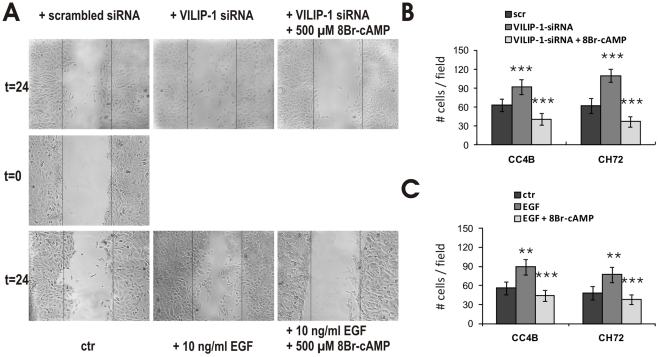
Involvement of cAMP-signaling in VILIP-1-siRNA- or EGF-induced migration of SCC cells. Confluent monolayers of VILIP-1-positive cells, CC4B and CH72, were wounded and the migratory capacity of the cells was measured within 24 hrs by counting the number of cells per field in at least 8 fields from three different experiments. **A**. Representative wounds in monolayers of EGF- or siRNA-treated CC4B cells are shown in comparison to untreated controls and to cells that were additionally treated with 8Br-cAMP (500 µM) 24 h before wounding. **B**. Quantification of the migration-inducing effect of siRNA knock down of VILIP-1 with or without application of 8Br-cAMP in CC4B and CH72 cells (p<0.001 in all conditions). **C**. Quantification of the migration-inducing effect of EGF-treatment with and without application of 8Br-cAMP (p<0.001 in all conditions). Data represent mean values from at least three independent experiments and error bars indicate standard deviations. Asterisks indicate the level of significance.

To demonstrate the involvement of cAMP-signaling in the effect of EMT-induction and of VILIP-1-expression on the migratory capability of skin tumor cells, we performed *in vitro* wound closure assays. We either knocked down VILIP-1-expression by siRNA or applied EGF-stimulation leading to reduced VILIP-1-expression (Fig. 6). Both the knock down of VILIP-1-expression by siRNA and EGF treatment resulted in a significantly increased migratory capability (Fig. 6B), documented by a higher number of migrating cells in the wound area after 24 h (Fig. 6A). In CC4B cells VILIP-1-specific siRNA enhanced the cell migration by 46% (p<0.001) and EGF by 59% (p<0.001). In CH72 cells following siRNA treatment 72% more (p<0.001) and following EGF treatment 60% more (p<0.001) migrating cells were observed. We have previously shown that VILIP-1-negative SCCs show greater migratory capability than their VILIP-1-positive counterparts, and that this effect depends on decreased cAMP levels [18]. The application of 8Br-cAMP 24 h before wounding of siRNA or EGF treated cells prevented the enhancement of the migratory capability and significantly reduced the number of cells in the wound area (Fig. 6, A, B and C: p<0.001 in all conditions). Following the additional 8Br-cAMP application, the number of migrating cells was significantly lower than in control conditions (Fig. 6; B: CC4B: p = 0.009, CH72: p = 0.002; C: CC4B p = 0.037, CH72 p = 0.039), confirming that increased motility induced by the loss of VILIP-1 or by EGF treatment is suppressed by cAMP-signaling. These results point towards a role of the putative tumor migration suppressor VILIP-1 and the associated cAMP pathway for EMT in SCC.

## Discussion

In this study, we examined the role of the putative tumor invasion suppressor VILIP-1 and cAMP-signaling during EMT in mouse skin tumor cell lines of different aggressiveness. When aggressive, VILIP-1-negative SCCs were compared to less aggressive VILIP-1-positive SCCs, distinct differences in morphology were observed. These differences resemble the change in cellular morphology during the transition of the epithelial to mesenchymal phenotype. This assumption was further supported by the results of the Western blot analysis, showing the loss of the EMT-marker E-cadherin in VILIP-1-negative CC4A and CH72T3 cells. In addition, a previous study revealed increased activity of two further EMT markers, RhoA and MMP9, in the aggressive, VILIP-1 negative SCCs [18]. The spindle-like morphology, the loss of the epithelial marker gene E-cadherin together with the previously shown up-regulation in the activity of RhoA, MMP9 and of the protein level of integrin α5, as well as the enhanced migratory capability, indicate that VILIP-1-negative, aggressive SCCs underwent EMT, and that down-regulation of VILIP-1 might be related to EMT. To reproduce this process experimentally, we stimulated VILIP-1-positive CC4B and CH72 cells with EMT-inducing growth factors TGFβ and EGF. We found that stimulation with EGF induces SCC cells to undergo a transition from the epithelial to the spindle-like mesenchymal morphology. This was accompanied by the loss of E-cadherin and subsequent loss of cell-cell-contacts. Similar results have been obtained for several other carcinoma cells by authors of previous studies [29], [32], [33]. In addition EGF treatment leads to the up-regulation of integrin α5, and most importantly to the down-regulation of VILIP-1 in CC4B and CH72 cells. This affirms the hypothesis that VILIP-1 is lost during EMT. EGF treatment of CC4B and CH72 cells induced an EMT-like phenomenon and caused VILIP-1-positive SCC cells to mimick the characteristics of VILIP-1-negative SCC cells, including the gain of increased migratory capability. In the literature TGFβ was also shown to induce EMT [34], [35]. However, in CC4B and CH72 cells TGFβ caused rounding of cells, but not cell shape elongation, and only slightly reduced E-cadherin in CH72 cells or even increased it in CC4B cells. Such increased E-cadherin levels following TGFβ treatment have also been observed in human trophoblasts [36]. Another study revealed, that only 2 of 20 mouse cell lines treated with TGFβ responded with the induction of EMT [37]. In keratinocytes it has been shown, that the induction of EMT by TGFβ depends on a hyperactive Ras-MAPK-pathway and that without this prerequisite only reversible morphological alterations are induced [38]. However, the loss of growth control induced by TGFβ that occurs at a late stage of mouse skin carcinogenesis is independent of ras gene activation [36]. These findings might explain the small effect of TGFβ observed in this study. VILIP-1-expression was not or only marginally affected by TGFβ treatment. Thus, EGF, rather than TGFβ is a key factor in malignant progression of squamous cell carcinoma lines.

The observed down-regulation of E-cadherin and VILIP-1-expression during EGF-induced EMT might be caused either by a parallel transcriptional repression of both genes or by a serial mechanism, where reduced levels of VILIP-1/cAMP might contribute in a second step to the down-regulation of E-cadherin. We have previously shown that reduced VILIP-1/cAMP levels contribute to the up-regulation of integrin α5 in mouse skin SCC [19]. Therefore, we analyzed the expression of Snail1, as a potent inducer of EMT and a transcriptional repressor of E-cadherin. Snail1 was detectable in untreated aggressive, VILIP-1- and E-cadherin-negative SCC cells and was inducible by EGF treatment in less aggressive, VILIP-1-positive cells. These results suggest the possible involvement of Snail1 in the repression of E-cadherin and VILIP-1-expression during EMT of mouse skin SCC. The inverse correlation of Snail1 and E-cadherin expression, together with the up-regulation of Snail1 during EGF-induced EMT are in line with findings from other studies investigating the characteristics of invasive SCC [29], [39], [40]. It is widely believed that downstream pathways of the EGFR, particularly the PI3K-Akt and MAPK pathway, are involved in the initiation of Snail1 expression through the regulation of NF-κB and AP-1, which act as transcriptional activators of the Snail1 gene [8], [40], [41]. It is noteworthy that in our study the enhancement of cAMP levels by application of forskolin in EGF treated cells repressed EGF-induced expression of Snail1, implicating cAMP-signaling in the regulation of Snail1. Ectopic expression of VILIP-1 in aggressive, VILIP-1-negative SCCs, which leads to increased cAMP levels [18], likewise decreased the expression level of Snail1. This effect could be blocked by the application of the general adenylyl cyclase inhibitor DDA. To our knowledge this is the first study showing that VILIP-1-dependent cAMP-signaling interferes with the expression of Snail1 and might thereby prevent the progression of EMT during tumor progression. Accumulating evidence indicates that enhanced cAMP-signaling counteracts the malignant progression of cancer cells [42], [43]. A few studies also report that cAMP-elevating agents block EMT [44]–[46]. In melanoma cells cAMP regulates the NF-κB-mediated expression of EMT-associated genes [44]. Among these genes were SIP1 and slug, two other repressors of E-cadherin expression. In the alveolar epithelial cell line A549 increased cAMP levels resulting from the inhibition of cAMP-PDE4 block TGFβ-induced EMT in a MAPK-signaling dependent manner [46]. The two latter studies also describe a cAMP-mediated regulation of E-cadherin expression, whereas in other studies analyzing E-cadherin-mediated cell-cell-contacts and migration of cancer cells no cAMP-dependent effect on E-cadherin expression could be detected [11], [47]. Although we found a significant effect of VILIP-1 and cAMP-signaling on the expression level of the E-cadherin repressor Snail1, we could not detect any effect, neither of the knock down of VILIP-1 in the less aggressive SCCs, nor of the over-expression of VILIP-1 in the aggressive SCCs, on the expression of E-cadherin. Thus, we conclude that VILIP-1 is not necessary for basal expression of E-cadherin. Ectopic expression of VILIP-1 and subsequently increased cAMP levels seem not to be sufficient to abolish an established inactivation of the E-cadherin gene. Therefore, E-cadherin and VILIP-1 are rather subject to a parallel transcriptional repression during EGF-induced EMT in mouse skin SCC. E-cadherin silencing involves a high degree of complexicity with the cooperation of epigenetic mechanisms and different repressors acting at different stages of the malignant progression [8]. Against this background it has to be considered that the Snail1-reducing effect of VILIP-1-cAMP might have an impact on the initial down-regulation of E-cadherin expression during the first steps of tumor progression, whereas in advanced stages the contribution of additional factors is necessary to reconstitute the E-cadherin expression. However, it is very likely that VILIP-1 and cAMP-signaling regulates other Snail1 repressor target genes during EMT. To understand this interesting phenomenon further studies are required to decipher the precise mechanism of the VILIP-1-cAMP-dependent Snail-1 regulation and its impact on gene repression. For instance, the reduction of integrin α5β1 signaling by VILIP-1/cAMP might be involved, since integrin α5β1 was shown to act in concert with the EGFR and via ILK-Akt-NF-κB signaling, which constitute two ways to influence the expression level of Snail1 [8], [41], [48]. Another way to interfere with the induction of the EMT program and Snail1 expression is the direct crosstalk of cAMP-signaling with the signaling cascades downstream of the EGFR, such as interfering with the MAPK cascade on the level of Raf or with PI3K pathways on the level of GSK3β and NF-κB activity [44], [49], [50].

Further evidence for the EMT-suppressing role of VILIP-1-cAMP-signaling comes from the *in vitro* wound closure assays. The increase in the migratory capability of less aggressive, VILIP-1-positive SCCs caused by either siRNA knock down of VILIP-1 or EGF-treatment was eliminated by the application of 8Br-cAMP. Other studies analyzing the effects of EGF on cell migration, consistently describe an increase in the migratory capability following EGF treatment [31], [42]. As mentioned above the role of cAMP in tumor progression is controversial. For example, dibutyryl cAMP has been shown to slightly enhance collagen-mediated keratinocyte migration [51]. In contrast, it has also been shown that cAMP inhibits growth factor-mediated matrix metalloproteinase 9 induction and keratinocyte migration [42]. We have reported in a previous study that in mouse skin SCC enhanced cAMP-signaling reduced their migratory capability [22]. Accordingly, the results of the present study showed that the migration-diminishing effect of cAMP-signaling counteracts the migration-inducing effect of EGF, suppressing a further hallmark of malignant tumors cells, which have undergone EMT.

In summary, the present study shows the role of the putative tumor migration suppressor VILIP-1 in counteracting the induction of EGF-induced EMT. Our finding that VILIP-1 suppresses the expression of the EMT-related transcriptional repressor Snail1, and might thereby interfere with the induction of EMT in a cAMP-dependent manner, suggests a novel mechanism for the anti-invasive activity of VILIP-1-cAMP-signaling. Therefore, further investigation of the signaling networks involved in the VILIP-1-cAMP-mediated regulation of Snail1 and its targets in malignant tumors may help to identify novel anti-cancer strategies.
