# Correction: The putative Tumor Suppressor VILIP-1 Counteracts Epidermal Growth Factor-Induced Epidermal-Mesenchymal Transition in Squamous Carcinoma Cells

**DOI:** 10.1371/annotation/9453b87a-098a-44ee-9e76-97eebb50b641

**Published:** 2013-12-31

**Authors:** Katharina Schönrath, Andres J. Klein-Szanto, Karl H. Braunewell

Figures 1B and 5A in the article are generated using bands from different blots. The VILIP-1 expression in Figure 1B is taken from a consecutive blot as shown in the accompanying raw data file. In Figure 5A the PCR bands for Snail1 in CC4A, CH72T3, CC4B, and CH72 cells, respectively, were rearranged to match the loading design of the PCRs for GAPDH and VILIP-1 as shown in the accompanying raw data file.

Figure 1 can be viewed here: http://plosone.org/corrections/pone.0033116.g001.cn.tif

and its raw data file can be viewed here: http://plosone.org/corrections/pone.0033116.g001.raw.tif 

Figure 5 can be viewed here: http://plosone.org/corrections/pone.0033116.g005.cn.tif

and its raw data file can be viewed here: http://plosone.org/corrections/pone.0033116.g005.raw.tif

The revised figure legends for these two figures should read as follows:

Fig. 1. EMT-related differences in the characteristics of VILIP-1-positive and VILIP-1-negative SCC. A. Differences in morphology and in the formation of cell-cell contacts between VILIP-1-negative, aggressive CC4A and CH72T3 cells and the VILIP-1-positive, less aggressive CC4B and CH72 cells. B. Western Blot analysis showing the reciprocal expression levels of the adhesion molecules E-cadherin and integrin α5 in the VILIP-1-negative and -positive cell lines. As control for protein loading the β-actin levels were examined. Note, Fig. 1B is a composed arrangement with bands for VILIP-1 expression taken from a consecutive blot (see raw data file).

Fig. 5. The effect of modulation of VILIP-1-expression and cAMP-signaling on Snail1 mRNA levels. A. RT-PCR analysis showing the suppression of Snail1 mRNA by ectopic expression of VILIP-1 in VILIP-1-negative, aggressive CC4A and CH72T3 cells (lanes 2, 3 and 5, 6) and the blocking of this effect by application of DDA (500 µM) (lanes 3, 6). siRNA knock down of VILIP-1 in VILIP-1-positive less aggressive CC4B and CH72 cells caused no alteration of the expression level of Snail1 (lanes 8, 10). B. Densitometry of RT-PCR analysis in A, lanes 1 to 6. Intensity of Snail1 bands was normalized to the intensity of GAPDH control PCR bands (CC4A + VILIP-1: p = 0.035, CH72T3 +VILIP-1 p= 0.037). Bars represent the mean of three experiments. Error bars indicate standard deviations. Asterisks indicate the level of significance. Note, Fig. 5A is a composed arrangement with PCR bands for Snail1 rearranged to match the loading design of the PCRs for GAPDH and VILIP-1 (see raw data file).

